# 

**Published:** 1920-10-23

**Authors:** 


READY IMMEDIATELY.

BURDETT'S

HOSPITALS

AND

CHARITIES

The Year-Book of Philanthropy and Hospital Annual.

1920.

Price 17/6 net. By Post 18/6

Orders should be sent at once as the Edition is limited.

THE SCIENTIFIC PRESS, LTD.,
28/29 Southampton Street,
Strand, - London, W.C. 2.

THE SCIENTIFIC PRESS PUBLICATIONS.

PHOTOMICROGRAPHY.

By Edmund J. Spitta, L.R.C.P. Lond., M.R.C.S. Eng., F.R.A.S. Size 11 in. by 8½ in. With 41 Half-tone Reproductions from original Negatives and 63 Illustrations in the Text. Cloth, gilt. — 15s. net. 16s. Post free.

PRACTICAL MEDICAL ELECTRICITY and RADIOGRAPHY.

A Handbook for House Surgeons and Practitioners. By Alfred C. Norman, M.D. (Edin.). Size 7½ in. by 5 in. Profusely Illustrated. Cloth, gilt. — 5s. 6d. net. 6s. Post free.

HUMAN TEMPERAMENTS: STUDIES in CHARACTER.

By Chas. Mercier, M.D., F.R.C.P. Size 7½ in. by 5 in. New Edition, revised. Paper covers. — 1s. 3d. net. 1s. 4½d. Post free.

A VISION of STATE MEDICAL SERVICE.

By Col. G. T. K. Maurice, C.M.G. — 1s. net. 1s. 1½d. Post free.

THE SCIENTIFIC PRESS, LTD.,
28/29 Southampton St., Strand, London, W.C. 2.

### Matrons' and Sisters' Appointments.

James Hopkins Memorial Home, Ryton-on-Tyne.—Miss Margherita Mitchell has been appointed sister. She was trained at St. George's Union Infirmary, where she was later staff nurse, and subsequently home sister at the Northumberland War Hospital and the Infectious Diseases Hospital, Walker Gate, Newcastle-on-Tyne.

Perth Royal Infirmary.—Miss Mary Christie has been appointed theatre sister, and Miss E. C. Murray sister of the surgical wards. Miss Christie was trained at the Royal Infirmary, Dundee, and Miss Murray at the Royal Infirmary, Edinburgh, and the City Hospital, Edinburgh, later holding the post of sister at Kirkcaldy Hospital.

Royal Chest Hospital, City Road.—Miss Mary Loughlin has been appointed visiting sister. She was trained at the Royal Salop Infirmary, Shrewsbury, has been staff nurse at St. Bartholomew's Hospital, sister at the National Hospital, Queen's Square, and the Royal Chest Hospital, sister-in-charge of the Coatham Convalescent Home, Redcar, and at Maltings Farm Sanatorium, Nayland, and for five years was sister at the 1st Eastern General Hospital, Cambridge.

Royal National Orthopædic Hospital.—Miss Maud Mann has been appointed sister. She was trained at the West London Hospital, Hammersmith, and the Children's Hospital, Gloucester. She has been sister at the General Hospital, Chiswick, and temporary assistant matron at the Hostel of St. Luke, Fitzroy Square.

Kilmarnock Infirmary.—Miss Catherine G. Tyson has been appointed night sister. She was trained at Hull Royal Infirmary and Monsall Fever Hospital, Manchester, later holding the appointment of sister at the Blackburn Royal Infirmary and sister under the Q.A.I.M.N.S.R.

Essex County Hospital, Colchester.—Miss M. Van Rompacy and Miss Jean Brown have been appointed night and theatre sisters respectively. Both were trained at the Essex County Hospital, where Miss Brown has since been sister, first of a military and then of a men's medical ward. Miss Van Rompacy, after her training, became staff nurse of the British Hospital, Paris, and later masseuse under the Ministry of Pensions, Dundee. She holds the certificate of the I.S.T.M.

Monsall Fever Hospital, Manchester.—Miss Kathleen Wingrove has been appointed sister. She was trained at the Great Northern Central Hospital.

Teignmouth Hospital, South Devon.—Miss Marjory M. Hatton has been appointed matron. She was trained at Bristol Royal Infirmary, has been ward, night, and home sister, and matron's assistant at East Sussex Hospital, Hastings, matron of Lyme Regis Hospital, and matron of Axminster Cottage Hospital.

### Gifts and Bequests.

The residue of the property of Professor Pitt Cobbett has been left to the East London Hospital for Children.

Mr. W. T. Rogers, of Birkenhead, has left £50 to the Home for Epileptics, Maghull.

The Alexandra Day Committee have made a grant of £200 to the Royal Hospital and Home for Incurables, Putney.

Lord Inchcape's Fund for King George's Sanatorium in connection with the Seamen's Hospital Society now amounts to £74,464.

Mr. James Bull, of Albion Road, Clapham, left £500 each to the Hospital for Sick Children, the East London Hospital for Children, and the Royal National Orthopædic Hospital.

